# Supplementary material for: Non-equilibrium dynamics of spin-lattice coupling
Source: Nat Commun. 2023 Nov 27;14:7778. doi: 10.1038/s41467-023-43581-9 (PMC10681982; doi:10.1038/s41467-023-43581-9)
Supplement: Supplementary file 1 — Supplementary Information [file 41467_2023_43581_MOESM1_ESM.pdf]

## I Tr-XRD on other reflections

Figure S1 displays the time trace and its FFT spectrum of the (1 3 19) reflection (Figs. S1**a** and S1**b**) and those of the (1 -1 41) reflection (Figs. S1**c** and S1d), which also show dynamics triggered by the THz pulse. There is a clear peak at ~2 THz in the FFT spectrum of the (1 3 19) diffraction intensity. This mode is attributed to an infrared-active phonon mode for $E_{\mathrm{THz}}$ polarized in the basal plane, as reported previously for a Y-type hexaferrite with similar chemical composition as our crystal [1]. On the other hand, the Fourier transform of the dynamics of the (1 -1 41) diffraction intensity shows a peak at around the electromagnon resonance frequency.

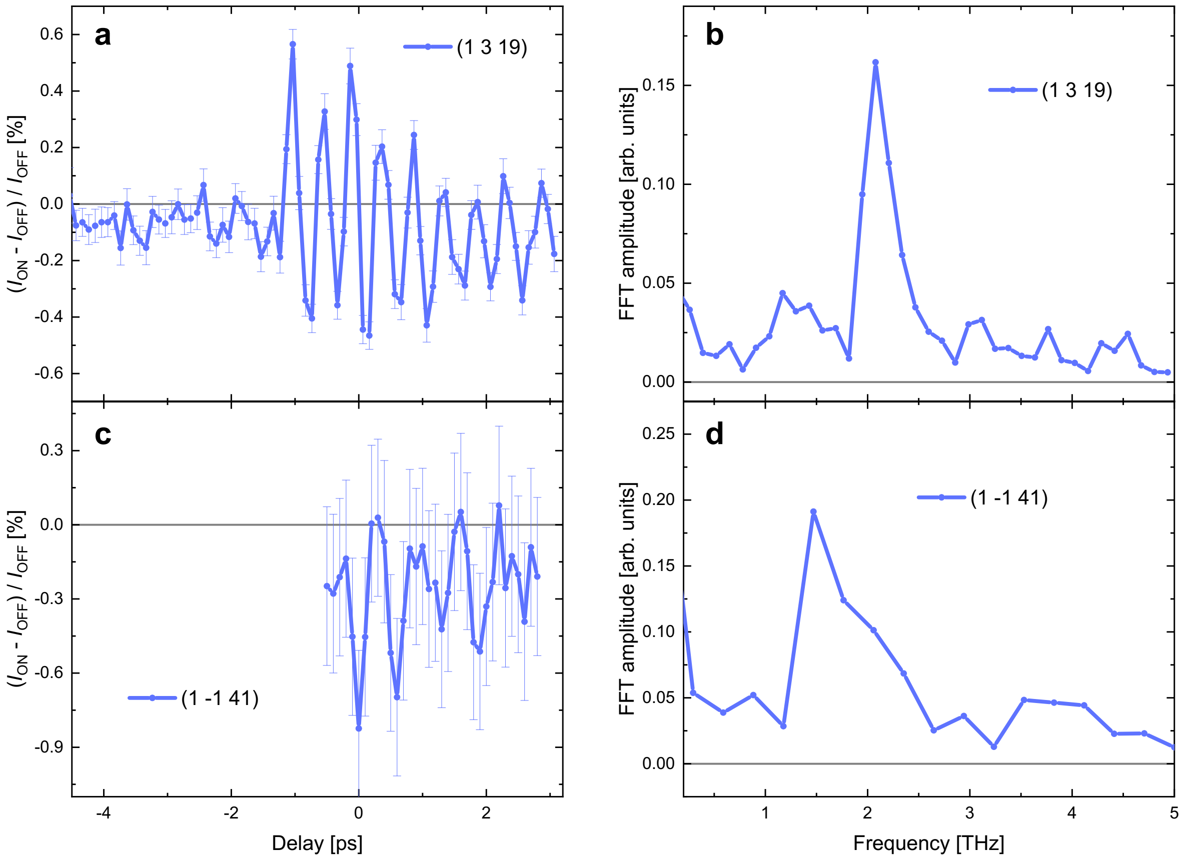


**Fig. S1 | tr-XRD signals. a** [**c**], The (1 3 19) [(1 -1 41)] X-ray diffraction intensity at 20 K as a function of pump-probe delay, and **b** [**d**], the FFT spectrum of the diffraction response. We used the low-pass THz filter for these measurements.

## II The Lorenz model to describe the resonant behavior

In a classical treatment, the lattice dynamics of zero-wavevector optical modes interacting with a spatially uniform electromagnetic field can be approximated by $N$ damped infrared-active harmonic oscillators that each follow the equation of motion

$$\frac{d^2 Q_n}{dt^2} + \gamma_n \frac{dQ_n}{dt} + {\omega_n}^2 Q_n = \sum_{\alpha ij} Z^*_{\alpha ij}\varepsilon_{n\alpha i}E_j(t). \quad (1)$$

Here for the $n$-th oscillator, $Q_n$ is the generalized coordinate, $\gamma_n$ is the damping constant, $\omega_n$ is the natural angular frequency of the oscillator, $Z^*_{\alpha ij}$ are elements of the Born effective

charge tensor, $\varepsilon_{n\alpha i}$ are the elements of the eigenvector for basis atom $\alpha$, and $E_i(t)$ are the Cartesian elements of the applied time-dependent electric field. Taking the Fourier transform of both sides gives

$$\tilde{Q}_n(\omega) = \frac{1}{{\omega_n}^2-\omega^2+i\gamma_n\omega} \sum_{\alpha ij} Z^*_{\alpha ij}\varepsilon_{n\alpha i}\tilde{E}_j(\omega). \quad (2)$$

We now assume that the Fourier transform of the polarization density $\tilde{\mathbf{P}}(\omega)$ is determined by the displacements of ions. To first order we then have

$$\tilde{P}_j(\omega) = \sum_{n\alpha i} \frac{Z^*_{\alpha ij}\varepsilon_{n\alpha i}\tilde{Q}_n(\omega)}{V_{\mathrm{uc}}}, \quad (3)$$

where $V_{\mathrm{uc}}$ is the volume of a unit cell. Substituting in Eq. (2), we then have

$$\tilde{P}_j(\omega) = \sum_n \frac{1}{{\omega_n}^2-\omega^2+i\gamma_n\omega} \sum_{\alpha\beta ikl} Z^*_{\alpha ij}Z^*_{\beta kl}\varepsilon_{n\alpha i}\varepsilon_{n\beta k}\tilde{E}_l(\omega). \quad (4)$$

This relation defines the linear susceptibility tensor

$$\chi_{ij}(\omega) = \sum_n \frac{1}{{\omega_n}^2-\omega^2+i\gamma_n\omega} \sum_{\alpha\beta kl} Z^*_{\alpha ki}Z^*_{\beta lj}\varepsilon_{n\alpha k}\varepsilon_{n\beta l}. \quad (5)$$

Non-vibrational contributions to the susceptibility from e.g. electronic transitions can be included as an approximately frequency-independent contribution in the THz range.

From inspection of Eq. (2), we observe that when $\omega = \omega_n$, there is a π/2 phase shift of the dynamics of $Q_n$ from the applied electric field. This results in a phase shift of π/2 between the driving THz field and the tr-XRD intensities response from the modes with phononic character, as found in Fig. 2**e**. This is consistent with the direct resonant driving of the electromagnon in contrast to the resonant but indirect driving of the magnetic modulations shown in Fig. 3**c**, which has also been observed in $TbMnO_3$ [2].

## III. The spectrum of THz pulse for THz-TDS measurements

Figure S2 shows the THz pulse spectrum used for the THz-TDS experiment. In contrast to those used for the ultrafast X-ray diffraction experiments, there are almost no components above 3 THz.

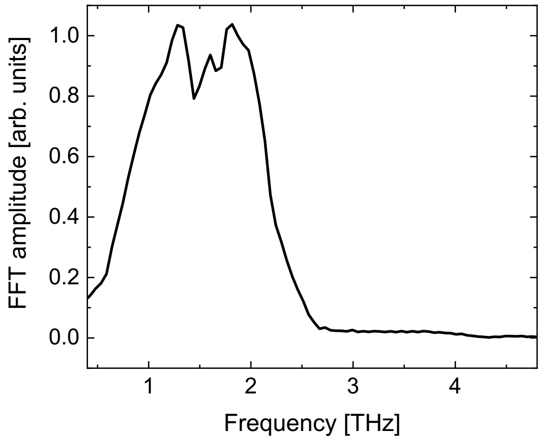


**Fig. S2 | THz pulse spectrum used for the THz-TDS experiment**. Fast-Fourier transform of the electric-field time trace of the THz pulses used for the THz time-domain spectroscopy experiments.

## IV Consideration of contributions to tr-SRXD signals other than sublattice magnetization dynamics

Although magnetic scattering is significantly enhanced at this atomic resonance, it is not the only channel that could contribute to the diffraction intensity. The X-ray scattering at an atomic resonance is represented by a second-rank tensor and involves three contributions: (i) charge scattering by electric monopoles (lattice), (ii) magnetic scattering by magnetic dipoles (magnetism), and (iii) orbital scattering by electric quadrupoles (aspheric charge distribution due to orbital hybridization with ligands). The last can only contribute to reflections whose indices are integers (reciprocal points), and therefore does not need to be further considered.

Lattice dynamics could in principle contribute to the dynamics of the (0 0 4.5) intensity either directly via charge scattering or indirectly via magnetic scattering: since the structure factor of a pure antiferromagnetic reflection is proportional to the phase factor of the resonant ion position, a time-dependent change in their positions results in a modulation of the magnetic diffraction intensity. To test the sensitivity of reflections accessible by soft X-ray scattering to vibrational motions induced by the THz pulse, we also measured the (0 0 3) Bragg diffraction intensity off-resonance, where only lattice dynamics can modulate the diffraction intensity. Figure S3 shows the time-resolved diffraction intensity of the (0 0 3) Bragg reflection off-resonance (650 eV) as a function of pump-probe delay. No clear response from the THz pulse is observed within our measurement precision. A possible contribution is also expected to be very small due to the low momentum transfer of reflections available in the soft X-ray regime. The different observed phase shifts between the tr-RSXD magnetic signals and the tr-XRD lattice signals (compare Figs. 2 and 3) also suggest that the tr-RSXD responses are not directly due to the lattice dynamics.

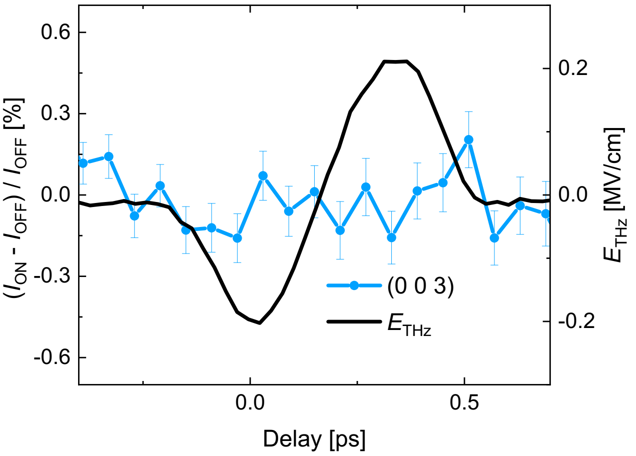


**Fig. S3 | Time-resolved diffraction signals of the (0 0 3) Bragg reflection off-resonance.** Blue points represent the (0 0 3) Bragg reflection intensity measured at 650 eV, while the black curve represents the incident E-field of the THz pulse measured with electro-optic sampling at the sample position (same as data as shown in Fig. 3**a**).

## V A model of the lattice vibration of the electromagnons

As described in the main text, displacements of the oxygen atoms at the border between two adjacent magnetic blocks may result in significant magnetic dynamics because of direct modulation in the magnetic frustration that stabilizes the conical magnetic order. Here, we assume a simplified model of the vibrational component of the electromagnon where the oxygen atoms at the borders move relative to the rest atoms by identical amounts along [001] (see Fig. S4**a**). We then estimate the amplitude of such displacements needed to cause a ~0.5% change in the (0 0 24) diffraction intensity (see Fig. 2**e**). Fig. S4**b** shows the normalized (0 0 24) diffraction intensity as a function of the displacements of the oxygen atoms at the interfaces between the blocks. We find that displacements of 0.32 pm result in the observed maximum diffraction intensity change. This amount of displacement in the oxygen atoms leads to a change in the bond angles that dominate magnetic frustration by ±0.15°, which is sufficiently large to change the relative angle between magnetic moments from two adjacent magnetic blocks [3]. Note that our calculation is based on the crystal structure of an analogue Y-type hexaferrite in the absence of the weak ferroelectric distortion [4]. It provides only a rough estimation because of the unknown exact crystal structure in the multiferroic phase of the material.

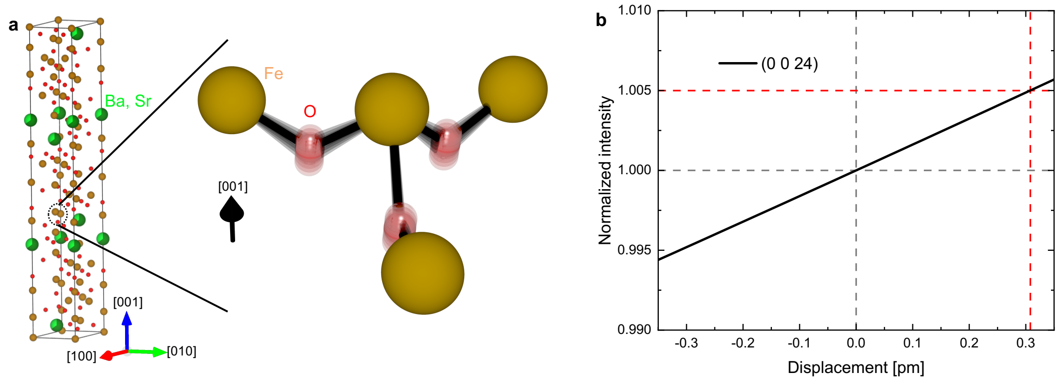


**Fig. S4 | Model of the lattice vibration in the electromagnons. a**, Crystal structure of a hexaferrite and enlarged view of the Fe-O-Fe bonds that dominate magnetic frustration. In the model described in the text, the oxygen atoms move along [001] while the excitation, which changes the bond angles and resultant magnetic frustration. As a result, the displacement of the oxygen atoms can affect the magnetic ground state. **b**, Calculated (0 0 24) Bragg reflection intensity as a function of the displacements of the oxygen atoms shown in **a**. Red broken lines indicate the point corresponding to the observed change in the tr-XRD experiments at 20 K.

## VI Calculation of magnetic diffraction intensity with the proposed spin-dynamics model

Here we estimate the amplitude of the spin dynamics by using the simplified model for the electromagnon in a Y-type hexaferrite proposed by Nakajima et al. [5]. The model is shown in Fig. 1**c**. Using a Cartesian coordinate system, where $x$ (// $M$) is along [100], $y$ is along [120], and $z$ is along [001], static/dynamic magnetic moments from each magnetic block are represented as shown in Table S1.

**Table S1 |** Magnetic moments in respective magnetic blocks, static and dynamic cases, together with the phase factor for the magnetic reflection at **Q** = (0, 0, 4.5). The latter is obtained by rotating a static magnetic moment by an angle $\Delta$ for an L block and $\Delta'$ for an S block along $y$. Here the total size of the magnetic moment is represented as $\mu_S$ for an S block with an open angle of the conical structure $\beta$ and $\mu_L$ for an L block with an open angle of the conical structure $\alpha$. Considering the preferential distribution of the divalent transition metals and $Al^{3+}$ into octahedral/tetrahedral sites [6-8], $\mu_S$ and $\mu_L$ are obtained as $4.1\mu_B$ and $15.8\mu_B$, respectively. The open angles of the conical structure $\alpha$ and $\beta$, which are a function of a magnetic field, can be estimated by the procedure shown in Sec. VII.

| Magnetic block | Static magnetic moment | Dynamic magnetic moment | Phase |
|---|---|---|---|
| S1 | $\mu_S\begin{pmatrix}-\cos\beta\\0\\\sin\beta\end{pmatrix}$ | $\mu_S\begin{pmatrix}-\cos(\beta+\Delta')\\0\\\sin(\beta+\Delta')\end{pmatrix}$ | 1 |
| L1 | $\mu_L\begin{pmatrix}\cos\alpha\\-\sin\alpha\\0\end{pmatrix}$ | $\mu_L\begin{pmatrix}\cos\alpha\cos\Delta\\-\sin\alpha\\-\cos\alpha\sin\Delta\end{pmatrix}$ | -*i* |
| S2 | $\mu_S\begin{pmatrix}-\cos\beta\\0\\-\sin\beta\end{pmatrix}$ | $\mu_S\begin{pmatrix}-\cos(\beta+\Delta')\\0\\-\sin(\beta+\Delta')\end{pmatrix}$ | -1 |
| L2 | $\mu_L\begin{pmatrix}\cos\alpha\\\sin\alpha\\0\end{pmatrix}$ | $\mu_L\begin{pmatrix}\cos\alpha\cos\Delta\\\sin\alpha\\\cos\alpha\sin\Delta\end{pmatrix}$ | *i* |

To test if the simplified model can explain the observed oscillations of the (0 0 4.5) magnetic reflection, we calculate the magnetic diffraction intensities as a function of the magnetic-moment angle deviation from equilibrium, $\Delta$ (L-block moments) and $\Delta'$ (S-block moments). The angle deviation concerns the $y$ direction (normal to the net magnetization axis and lying in the basal plane) based on the proposed electromagnon dynamics [5]. A resonant magnetic scattering by a single atom is represented as [9]

$$f_{\text{mag}} = -\frac{3}{4\pi q} i(\boldsymbol{\varepsilon}' \times \boldsymbol{\varepsilon}) \cdot \mathbf{m}_j (F_{-1}^1 - F_{+1}^1), \tag{6}$$

where $\boldsymbol{\varepsilon}$ and $\boldsymbol{\varepsilon}'$ are the polarization vector of the incident and scattered beams, respectively, $\mathbf{m}_j$ is the quantization axis of the magnetic moment at site $j$, $\mathbf{q}$ is the wave vector of the incident beam, and $F_{\pm 1}^1$ is the atomic properties of the dipole transition. The cross product of the polarization vectors are $\boldsymbol{\sigma}' \times \boldsymbol{\sigma} = \mathbf{0}$, $\boldsymbol{\sigma}' \times \boldsymbol{\pi} = \widehat{\mathbf{q}}$, $\boldsymbol{\pi}' \times \boldsymbol{\sigma} = \widehat{\mathbf{q}'}$, and $\boldsymbol{\pi}' \times \boldsymbol{\pi} = \widehat{\mathbf{q}'} \cdot \widehat{\mathbf{q}}$, where $\widehat{\mathbf{q}}$ ($\widehat{\mathbf{q}'}$) is the unit vector parallel to the incident (scattered) beam. Total diffraction intensity is represented by using the form factor for each polarization channel $M_{\varepsilon'\varepsilon}$ ($\varepsilon = \sigma$ or $\pi$), obtained by taking the sum of Eq. (6) over the whole crystal volume, as

$$I = \frac{1}{2}(|M_{\sigma'\sigma}|^2 + |M_{\sigma'\pi}|^2 + |M_{\pi'\sigma}|^2 + |M_{\pi'\pi}|^2) + P_2 \mathrm{Im}(M_{\sigma'\sigma}M_{\sigma'\pi}{}^* + M_{\pi'\sigma}M_{\pi'\pi}{}^*). \quad (7)$$

Here $P_2$ is the Stokes parameter that represents the degree of circular polarization. By using Table S1, the magnetic form factor at **Q** = (0, 0, 4.5) is obtained as

$$\mathbf{F}_m = \begin{pmatrix} 0 \\ 2i\mu_\mathrm{L}\sin\alpha \\ 2\mu_\mathrm{S}\sin(\beta+\Delta') + 2i\mu_\mathrm{L}\cos\alpha\sin\Delta \end{pmatrix}, \quad (8)$$

with the components for the channels being

$$M_{\sigma'\sigma} = 0, \quad (9)$$

$$M_{\sigma'\pi} = [2\mu_\mathrm{S}\sin(\beta+\Delta') + 2i\mu_\mathrm{L}\cos\alpha\sin\Delta]\sin\omega, \quad (10)$$

$$M_{\pi'\sigma} = -[2\mu_\mathrm{S}\sin(\beta+\Delta') + 2i\mu_\mathrm{L}\cos\alpha\sin\Delta]\sin(2\theta-\omega), \text{ and} \quad (11)$$

$$M_{\pi'\pi} = -2i\mu_\mathrm{L}\sin\alpha\sin 2\theta. \quad (12)$$

$\omega$ is the incident angle to the surface, and $2\theta$ is the scattered angle.

Figure S5 shows the intensity calculated by using Eqs. (7) and (9)-(12). The intensity is quadratic in $\Delta$ (see Fig. S5**b**) while almost linear in $\Delta'$ (see Fig. S5**a**). This means that if the motion of L-block moments is dominant for the electromagnon mode, we should observe twice the frequency of the electromagnon in the (0 0 4.5) magnetic diffraction time traces. On the other hand, the motion of S-block moments leads to oscillations with the electromagnon frequency. From Fig. S5**a** and 5**c**, we estimate an at least ~7° canting angle of the moments, which is even larger than the reported magnetic dynamics amplitude in $TbMnO_3$ [2], and up to a one-third change in $\Delta$ with respect to $\Delta'$.

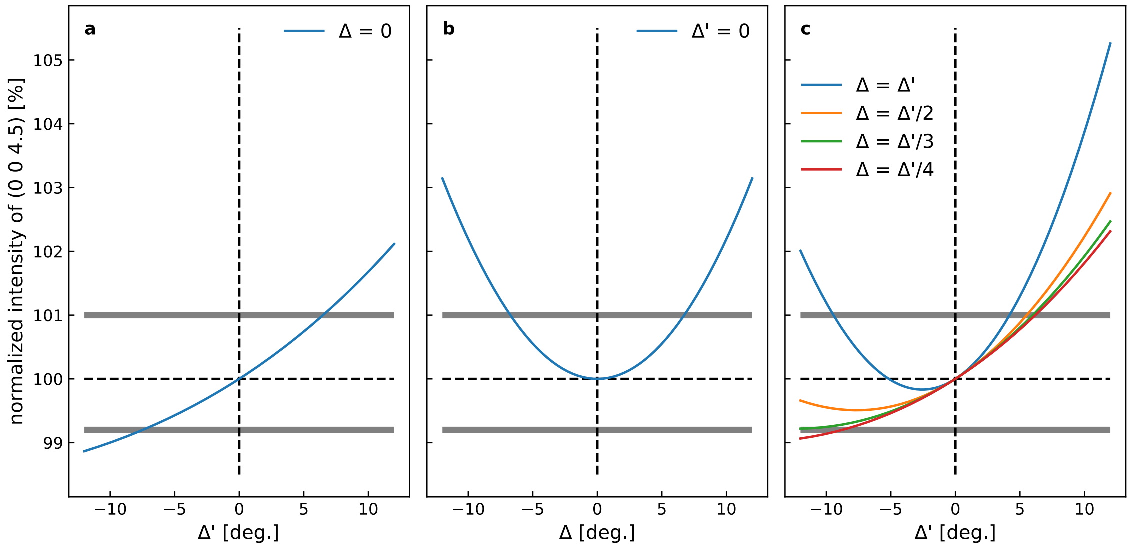


**Fig. S5** | Calculated (0 0 4.5) diffraction intensity as a function of the spin rotation angle $\Delta$ and/or $\Delta'$. **a**, **b**, For the case where only one of the sublattices (S-block moments or L-block moments) changes, and **c**, for the case where both sublattices change with different ratios.

## VII. An effective magnetic Hamiltonian

The energy the spin system acquires from the spin motion can be calculated from an effective magnetic Hamiltonian as the total energy difference between the ground state and the excited state. In this section, we introduce an effective magnetic Hamiltonian modeling the multiferroic hexaferrite. Our calculation is based on Refs. [10,11] showing an effective magnetic Hamiltonian modeling multiferroic hexaferrites given as

$$H = J_{\mathrm{LS}} \sum_{i,j=1,2} \boldsymbol{\mu}_{\mathrm{S}i} \cdot \boldsymbol{\mu}_{\mathrm{L}j} + 2J_{\mathrm{LL}} \boldsymbol{\mu}_{\mathrm{L1}} \cdot \boldsymbol{\mu}_{\mathrm{L2}} + 2J_{\mathrm{SS}} \boldsymbol{\mu}_{\mathrm{S1}} \cdot \boldsymbol{\mu}_{\mathrm{S2}} + D_{\mathrm{L}} \sum_j \left(\mu_{\mathrm{L}jz}\right)^2 + D_{\mathrm{S}} \sum_i (\mu_{\mathrm{S}iz})^2 - H_x \sum_{i,j=1,2} \left(\mu_{\mathrm{L}jx} + \mu_{\mathrm{S}jx}\right), \tag{13}$$

where $J_{\mathrm{LS}}$, $J_{\mathrm{LL}}$, and $J_{\mathrm{SS}}$ are the exchange interaction constants between an L and S blocks, between two L blocks, and between two S blocks, respectively, while $D_{\mathrm{L}}$ and $D_{\mathrm{S}}$ are the magnetic anisotropy constants along the *z* axis (// [001]) of an L block and S block, respectively. $H_x$ is a magnetic field (// *x*) that is the sum of the internal one ($H_{int}$) and external one ($H_{ext}$). Here we show how to determine these parameters as well as open angles of the conical structure $\alpha$ and $\beta$, partially referring to Ref. [10].

By using the static magnetic moments tabulated in Table S1, the equilibrium Hamiltonian is written as

$$H = -4J_{\mathrm{LS}}\mu_{\mathrm{L}}\mu_{\mathrm{S}} \cos\alpha \cos\beta + 2J_{\mathrm{LL}}{\mu_{\mathrm{L}}}^2 \cos 2\alpha + 2J_{\mathrm{SS}}{\mu_{\mathrm{S}}}^2 \cos 2\beta + 2D_{\mathrm{S}}{\mu_{\mathrm{S}}}^2 \sin^2\beta - 2(H_{int} + H_{ext})[\mu_{\mathrm{L}} \cos\alpha - \mu_{\mathrm{S}} \cos\beta]. \tag{14}$$

Representing the equilibrium $\alpha$ and $\beta$ as $\alpha_0$ and $\beta_0$, respectively, $\frac{\partial H}{\partial \alpha} = \frac{\partial H}{\partial \beta} = 0$ at $\alpha = \alpha_0$ and $\beta = \beta_0$. These conditions result in

$$2J_{\mathrm{LS}}\mu_{\mathrm{S}} \cos\beta_0 - 4J_{\mathrm{LL}}\mu_{\mathrm{L}} \cos\alpha_0 + (H_{int} + H_{ext}) = 0 \tag{15}$$

when $\sin\alpha \neq 0$, and

$$2J_{\mathrm{LS}}\mu_{\mathrm{L}} \cos\alpha_0 - 2(2J_{\mathrm{SS}} - D_{\mathrm{S}})\mu_{\mathrm{S}} \cos\beta_0 - (H_{int} + H_{ext}) = 0 \tag{16}$$

when $\sin\beta_0 \neq 0$. In a magnetic-field range $H_1 < H_{ext} < H_2$ (see Fig. S6), both Eqs. (15) and (16) need to be satisfied. A net magnetic moment $M$ [$\mu_{\mathrm{B}}$/f.u.] is expressed as

$$M = \mu_{\mathrm{L}} \cos\alpha_0 - \mu_{\mathrm{S}} \cos\beta_0 = \frac{2J_{\mathrm{LS}} - 2J_{\mathrm{LL}} - 2J_{\mathrm{SS}} + D_{\mathrm{S}}}{2[{J_{\mathrm{LS}}}^2 - 2J_{\mathrm{LL}}(2J_{\mathrm{SS}} - D_{\mathrm{S}})]}(H_{int} + H_{ext}). \tag{17}$$

In a magnetic-field range $H_2 < H_{ext}$ (see Fig. S6), $\alpha_0 = 0$ while $\beta_0 \neq 0$ [12]. In this case, by using Eq. (16) $M$ is expressed as

$$M = \mu_{\mathrm{L}} - \mu_{\mathrm{S}} \cos\beta_0 = \frac{2J_{\mathrm{SS}} - D_{\mathrm{S}} - J_{\mathrm{LS}}}{2J_{\mathrm{SS}} - D_{\mathrm{S}}}\mu_{\mathrm{L}} + \frac{1}{2(2J_{\mathrm{SS}} - D_{\mathrm{S}})}(H_{int} + H_{ext}). \tag{18}$$

Fitting a magnetization curve in two magnetic-field regions as a linear function of $H_{ext}$ [Eqs. (17) and (18)] provides $J_{\mathrm{LS}}$, $J_{\mathrm{LL}}$, $2J_{\mathrm{SS}} - D_{\mathrm{S}}$, and $H_{int}$.

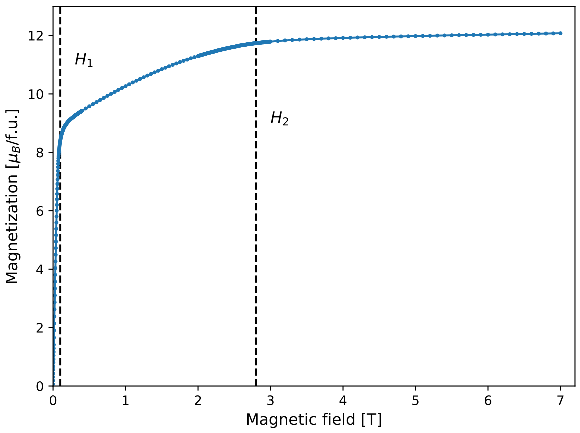


**Fig. S6 | In-plane magnetization**. The obtained in-plane magnetization of $Ba_{1.3}Sr_{0.7}CoZnFe_{11}AlO_{22}$ taken at 25 K. Vertical dashed lines indicate the two critical fields $H_1$ and $H_2$ defining the ranges used to extract the coefficients in the magnetic Hamiltonian.

By using the dynamic magnetic moments tabulated in Table S1, Eq. (13) becomes

$$H = -4J_{\mathrm{LS}}\mu_{\mathrm{L}}\mu_{\mathrm{S}}\cos\Delta\cos\alpha_0\cos(\beta_0+\Delta') + 2J_{\mathrm{LL}}{\mu_{\mathrm{L}}}^2(\cos^2\alpha_0\cos 2\Delta - \sin^2\alpha_0) + 2J_{\mathrm{SS}}{\mu_{\mathrm{S}}}^2\cos(2\beta_0+2\Delta') + 2D_{\mathrm{L}}{\mu_{\mathrm{L}}}^2\cos^2\alpha_0\sin^2\Delta + 2D_{\mathrm{S}}\,{\mu_{\mathrm{S}}}^2\sin^2(\beta_0+\Delta') - 2(H_{int}+H_{ext})[\mu_{\mathrm{L}}\cos\alpha_0\cos\Delta - \mu_{\mathrm{S}}\cos(\beta_0+\Delta')]. \quad (19)$$

As the magnetic ground state is at $\Delta = \Delta' = 0$, $\frac{\partial H}{\partial \Delta} = \frac{\partial H}{\partial \Delta'} = 0$ when $\Delta = \Delta' = 0$. This condition gives a relation between $\alpha_0$ and $\beta_0$,

$$2J_{\mathrm{LS}}\mu_{\mathrm{L}}\cos\alpha_0 - 2(2J_{\mathrm{SS}} - D_{\mathrm{S}})\mu_{\mathrm{S}}\cos\beta_0 - (H_{int}+H_{ext}) = 0. \quad (20)$$

For a given magnetization $M$ [$\mu_B$/f.u.], $\alpha_0$ and $\beta_0$ follow another relation

$$\mu_{\mathrm{L}}\cos\alpha_0 - \mu_{\mathrm{S}}\cos\beta_0 = M. \quad (21)$$

By using these equations, one can obtain the open angles of the conical structure.

The Landau-Lifshitz-Gilbert equation describes a precessional mode of magnetization as

$$\frac{d\boldsymbol{\mu}_{\mathrm{L}}}{dt} = -\gamma\boldsymbol{\mu}_{\mathrm{L}}\times(\nabla H) \text{ and} \quad (22)$$

$$\frac{d\boldsymbol{\mu}_{\mathrm{S}}}{dt} = -\gamma\boldsymbol{\mu}_{\mathrm{S}}\times(\nabla H) \quad (23)$$

for two sublattices, where $\boldsymbol{\mu}_{\mathrm{L}} = (\mu_{\mathrm{L}x},\mu_{\mathrm{L}y},0) + (\delta\mu_{\mathrm{L}x},\delta\mu_{\mathrm{L}y},\delta\mu_{\mathrm{L}z})$, $\boldsymbol{\mu}_{\mathrm{S}} = (\mu_{\mathrm{S}x},0,\mu_{\mathrm{S}z}) + (\delta\mu_{\mathrm{S}x},\delta\mu_{\mathrm{S}y},\delta\mu_{\mathrm{S}z})$, and damping terms being ignored for simplicity. Equations (22) and (23) are represented as

$$\begin{pmatrix} i\omega & 0 & -A & 0 & 0 & 0 \\ 0 & i\omega & -B & 0 & 0 & 0 \\ -C & -D & i\omega & -E & 0 & -F \\ 0 & 0 & 0 & i\omega & -G & 0 \\ -I & 0 & 0 & -K & i\omega & -L \\ 0 & 0 & 0 & 0 & -M & i\omega \end{pmatrix} \begin{pmatrix} \delta\mu_{\mathrm{L}x} \\ \delta\mu_{\mathrm{L}y} \\ \delta\mu_{\mathrm{L}z} \\ \delta\mu_{\mathrm{S}x} \\ \delta\mu_{\mathrm{S}y} \\ \delta\mu_{\mathrm{S}z} \end{pmatrix} = \mathbf{0}, \tag{24}$$

where $\omega$ is the eigenfrequency and see Ref. [10] for the definition of the coefficients ($A$, $B$, $C$, $D$, $E$, $F$, $G$, $I$, $K$, $L$, and $M$). Equation (24) gives a non-trivial solution when the determinant is zero. This condition leads to

$$\omega^2 = \frac{1}{2}\Big[-(AC + BD + GH + LM) \pm \sqrt{(AC + BD + GH + LM)^2 - 4(BDLM - AFIM + ACLM + BDGK - AEGI + ACGK)}\Big]. \tag{25}$$

Two electromagnon modes exist in a multiferroic hexaferrite [11]. By using $\omega_+ = 1.7$ THz (see Figs. 1**d** and 1**e**, or [1,5]) and $\omega_- = 41.7$ GHz reported previously [11], Eq. (25) provides $D_{\mathrm{L}}$ and $D_{\mathrm{S}}$.

## VIII Estimation of transferred energy between lattice and magnetic system

At first, we calculate the absorbed THz pulse energy into the hexaferrite sample. We assume a Gaussian profile of a monochromatic THz beam, whose electric-field component is represented as $E(t) = E_0 \mathrm{e}^{-t^2/4\tau^2}$, where $\tau$ is the pulse duration of the intensity in the root-mean-square and $E_0$ is the peak electric-field strength. From the electro-optic sampling data shown in Fig. 3**a**, $\tau$ and $E_0$ are ~0.20 ps and ~200 kV/cm, respectively. For the following discussion, we assume magnetic permeability as 1 since the contribution of magnetic permeability in the THz regime is known as negligibly small in a multiferroic hexaferrite showing electromagnon resonance at a similar frequency [13]. Transmission of light through the interface between a vacuum and the hexaferrite sample is given by the Fresnel equations

$$T = \frac{2n_1 \sin\theta_1}{n_2 \sin\theta_1 + n_1 \sin\theta_2}, \tag{26}$$

where $n_1$ and $n_2$ are the real part of the refractive index in a vacuum and the hexaferrite, respectively, and $\theta_1$ and $\theta_2$ are the incidence angle and the refraction angle to the sample surface, respectively. Snell's law gives $\theta_2 = 89.4°$ for $\theta_1 = 87.4°$ at the (0 0 4.5) magnetic reflection, where we use $n_2 = 4.25$ from Fig. 1**d** at 20 K. The energy density inside the sample just below the interface $I$ is

$$I = \frac{n_2 E(t)^2 T^2}{Z_0}, \tag{27}$$

where $Z_0$ is the optical impedance of vacuum. The energy absorbed into the formula unit at the surface $p$ (one S block and one L block, the area of $\sqrt{3}a^2/2$ and the depth of $c/3$) is

$$p = \frac{n_2 {E_0}^2 T^2}{Z_0} \frac{\sqrt{3}a^2}{2} \int_{-\infty}^{\infty} \mathrm{e}^{-t^2/2\tau^2} dt \, \frac{\int_0^{c/3} \mathrm{e}^{-z/\xi} dz}{\int_0^{\infty} \mathrm{e}^{-z/\xi} dz}. \tag{28}$$

Here $\xi$ is the penetration depth of the THz beam (~10 μm see Method). Equation (28) gives us a rough estimation of the THz energy transferred into the lattice as ~89 μeV/f.u.

As the second step, we estimate the energy that the magnetic system takes through spin-lattice coupling. As discussed in Sec. VI., the observed magnetic dynamics are 7° – 10° change in $\Delta'$ and up to one-third for $\Delta$ with respect to $\Delta'$. Putting this transient magnetic structure into the effective magnetic Hamiltonian Eq. (13) gives rise to 12 – 300 μeV/f.u. energy cost from the ground state, as shown in Fig. S7. Note that the absorbed THz energy must be the upper limit of the energy in the magnetic system, and hence, the energy that the magnetic systems take is in the same order of the absorbed THz energy. Even though our estimation is quite rough, efficient energy transfer in the spin-lattice coupling is indicated at an electromagnon resonance.

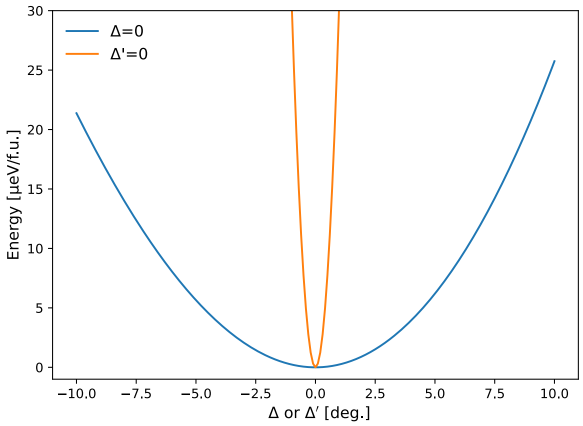


**Fig. S7 | Energy of a magnetic state.** Calculated energy differences for the spin rotation angles $\Delta$ or $\Delta'$ from the ground state ($\Delta = \Delta' = 0$), in cases of $\Delta$=0 (blue) and $\Delta'$=0 (orange).

## IX <u>Static characterization of the sample</u>

Figure S8 shows the (0 1 41) reflection profile along $L$, obtained at the Material Science beamline of the Swiss Light Source, Switzerland, as the equilibrium characterization of the sample measured at the SwissFEL. The profile confirms the good quality of the sample.

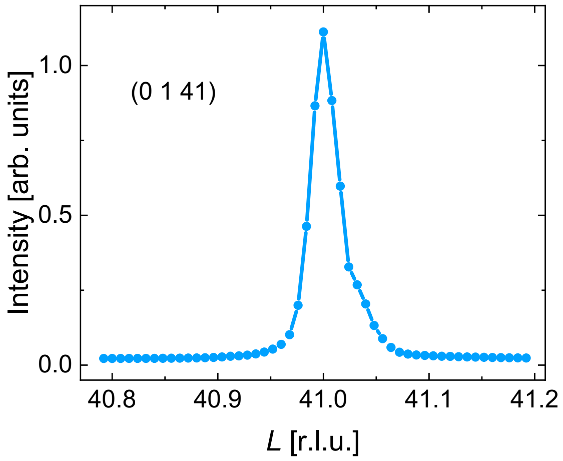


**Fig. S8 | Static characterization of the Y-type hexaferrite sample with hard X rays**. A diffraction profile around the (0 1 41) Bragg reflection along $L$.

Figure S9**a** shows an X-ray absorption spectrum around the Fe $L_3$ edge, measured on the sample prior to the time-resolved soft X-ray magnetic diffraction experiments, using an avalanche photodiode. Based on this measurement, the photon energy for the tr-RSXD measurements was set to match the clear resonant feature at 711 eV. A rocking curve around the (0 0 4.5) magnetic reflection is shown in Fig. S9**b**.

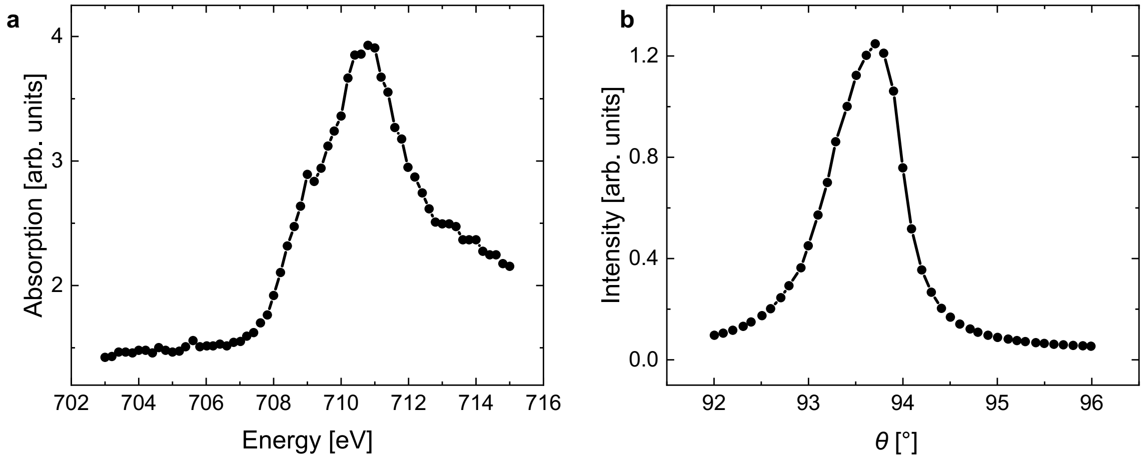


**Fig. S9 | Static characterization of the Y-type hexaferrite sample with soft X rays**. **a**, X-ray absorption spectrum around the Fe $L_3$ edge, and **b**, rocking curve around the (0 0 4.5) magnetic reflection.

## X Experimental geometry

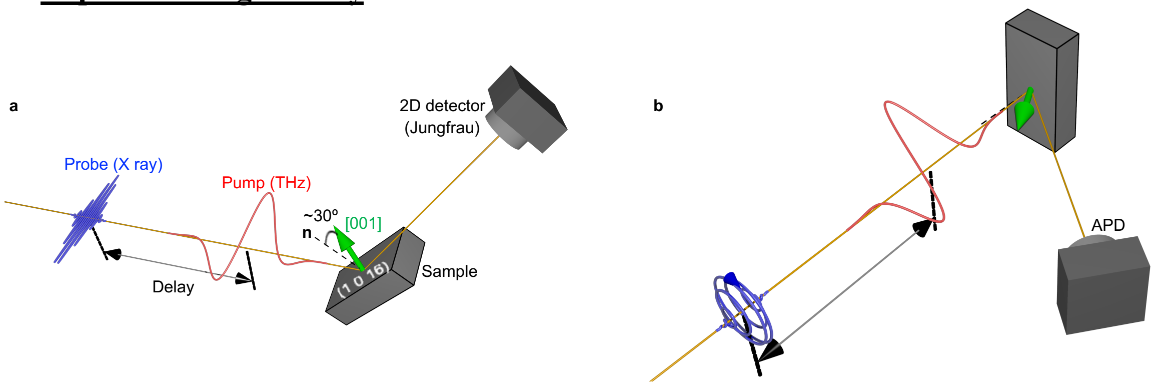


**Fig. S10 | Schematic view of the experimental setups for the time-resolved X-ray experiments.** **a**, For tr-XRD and **b**, for tr-RSXD. The [001] direction has an angle of ~30° from the surface normal **n**.

Supplementary References


[1] Vít, J., Kadlec, F., Kadlec, C., Borodavka, F., Chai, Y. S., Zhai, K., Sun, Y., & Kamba, S., Electromagnon in the Y-type hexaferrite $BaSrCoZnFe_{11}AlO_{22}$. *Phys. Rev. B*. **97**, 134406 (2018).

[2] Kubacka, T., Johnson, J. A., Hoffmann, M. C., Vicario, C., de Jong, S., Beaud, P., Grübel, S., Huang, S.-W., Huber, L., Patthey, L., Chuang, Y.-D., Turner, J. J., Dakovski, G. L., Lee, W.-S., Minitti, M. P., Schlotter, W., Moore, R. G., Hauri, C. P., Koohpayeh, S. M., Scagnoli, V., Ingold, G., Johnson, S. L., & Staub, U., Large-amplitude spin dynamics driven by a THz pulse in resonance with an electromagnon. *Science* **343**, 1333-1336 (2014).

[3] Utsumi, S., Yoshiba, D., & Momozawa, N., Superexchange interactions of $(Ba_{1-x}Sr_x)_2Zn_2Fe_{12}O_{22}$ system studied by neutron diffraction. *J. Phys. Soc. Jpn.* **76**, 034704 (2007).

[4] Momozawa, N., Neutron diffraction study of helimagnet $(Ba_{1-x}Sr_x)_2Zn_2Fe_{12}O_{22}$. *J. Phys. Soc. Jpn.* **55**, 4007-4013 (1986).

[5] Nakajima, T., Takahashi, Y., Kibayashi, S., Matsuda, M., Kakurai, K., Ishiwata, S., Taguchi, Y., Tokura, Y., & Arima, T., Electromagnon excitation in the field-induced noncollinear ferrimagnetic phase of $Ba_2Mg_2Fe_{12}O_{22}$ studied by polarized inelastic neutron scattering and terahertz time-domain optical spectroscopy. *Phys. Rev. B* **93**, 035119 (2016).

[6] Momozawa, N., Yamaguchi, Y., Takei, H., & Mita, M., Magnetic structure of $(Ba_{1-x}Sr_x)_2Zn_2Fe_{12}O_{22}$ ($x$ = 0-1.0). *J. Phys. Soc. Jpn.* **54**, 771-780 (1985).

[7] Mahmood, S. H., Jaradat, F. S., Lehlooh, A.-F., & Hammoudeh, A., Structural properties and hyperfine interactions in Co-Zn Y-type hexaferrites prepared by sol-gel method. *Ceram. Int.* **40**, 5231-5236 (2014).

[8] Kim, J., Choi, H., & Kim, C. S., Magnetic properties of polycrystalline Y-type hexaferrite $Ba_{2-x}Sr_xNi_2(Fe_{1-y}Al_y)_{12}O_{22}$. *AIP Advances* **10**, 015204 (2020).

[9] Hannon, J. P., Trammell, G. T., Blume, M., and Gibbs, D., X-ray resonance exchange scattering. *Phys. Rev. Lett.* **61**, 1245-1248 (1988).

[10] Chun, S. H., Shin, K. W., Kim, H. J., Jung, S., Park, J., Bahk, Y.-M., Park, H.-R., Kyoung, J., Choi, D.-H., Kim, D.-S., Park, G.-S., Mitchell, J. F., & Kim, K. H., Electromagnon with sensitive terahertz magnetochromism in a room-temperature magnetoelectric hexaferrite. *Phys. Rev. Lett.* **120**, 027202 (2018).

[11] Jang, H., Ueda, H., Kim, H.-D., Kim, M., Shin, K.-W., Kim, K.-H., Park, S.-Y., Shin, H. J., Borisov, P., Rosseinsky, M. J., Jang, D., Choi, H., Eom, Intae, Staub, U., & Chun, S. H., 4D visualization of the photoexcited coherent magnon by an X-ray free electron laser. *Adv. Mat.* **35**, 2303032 (2023).

[12] Lee, H. B., Song, Y.-S., Chung, J.-H., Chun, S. H., Chai, Y. S., Kim, K. H., Reehuis, M., Prokeš, K., & Mat'aš, S., Field-induced incommensurate-to-commensurate phase transition in the magnetoelectric hexaferrite $Ba_{0.5}Sr_{1.5}Zn_2(Fe_{1-x}Al_x)_{12}O_{22}$. *Phys. Rev. B* **83**, 144425 (2011).

[13] Kida, N., Kumakura, S., Ishiwata, S., Taguchi, Y., & Tokura, Y., Gigantic terahertz magnetochromism via electromagnons in the hexaferrite magnet $Ba_2Mg_2Fe_{12}O_{22}$. *Phys. Rev. B* **83**, 064422 (2011).
